# Impact of Mutations in *Arabidopsis thaliana* Metabolic Pathways on Polerovirus Accumulation, Aphid Performance, and Feeding Behavior

**DOI:** 10.3390/v12020146

**Published:** 2020-01-27

**Authors:** Florent Bogaert, Aurélie Marmonier, Elodie Pichon, Sylvaine Boissinot, Véronique Ziegler-Graff, Quentin Chesnais, Claire Villeroy, Martin Drucker, Véronique Brault

**Affiliations:** 1 Université de Strasbourg, Institut National de Recherche en Agriculture, Alimentation et Environnement, SVQV UMR-A1131, 68000 Colmar, France; 2 Institut de biologie moléculaire des plantes, Centre National de la Recherche Scientifique, Université de Strasbourg, 67084 Strasbourg, France

**Keywords:** polerovirus, *Arabidopsis thaliana* mutant, aphid biomass, electropenetrography (EPG), transmission

## Abstract

During the process of virus acquisition by aphids, plants respond to both the virus and the aphids by mobilizing different metabolic pathways. It is conceivable that the plant metabolic responses to both aggressors may be conducive to virus acquisition. To address this question, we analyze the accumulation of the phloem-limited polerovirus *Turnip yellows virus* (TuYV), which is strictly transmitted by aphids, and aphid’s life traits in six *Arabidopsis thaliana* mutants (*xth33*, *ss3-2*, *nata1*, *myc234*, *quad*, *atr1D,* and *pad4-1*). We observed that mutations affecting the carbohydrate metabolism, the synthesis of a non-protein amino acid and the glucosinolate pathway had an effect on TuYV accumulation. However, the virus titer did not correlate with the virus transmission efficiency. Some mutations in *A.* *thaliana* affect the aphid feeding behavior but often only in infected plants. The duration of the phloem sap ingestion phase, together with the time preceding the first sap ingestion, affect the virus transmission rate more than the virus titer did. Our results also show that the aphids reared on infected mutant plants had a reduced biomass regardless of the mutation and the duration of the sap ingestion phase.

## 1. Introduction

Aphids are a large group of Hemipteran insects that induce direct damage on plants but are, more importantly, major vectors of plant viruses. Among aphids, *Myzus persicae*, the green peach aphid, can transmit more than 100 different virus species to a variety of plant species including the model plant *Arabidopsis thaliana* [1]. The success of transmission results from molecular interactions between the virus, the plant, and the vector. Indeed, once an aphid probes and then feeds on a host plant, it releases salivary secretions that are recognized and elicit plant defenses [2,3,4,5]. These reactions implement a number of metabolic and physiological modifications that may have a detrimental effect on aphids [6,7,8,9]. Several hormonal pathways induced in reaction to *M. persicae* are characterized in *A. thaliana* and involve ethylene, salicylic, jasmonic, and abscisic acid-signaling pathways [7,8]. Secondary metabolites are also important in plant defense against aphids. For example, PAD4 (phytoalexin-deficient 4) and PAD3, a cytochrome P450 involved in the synthesis of camalexin, both act as key players in modulating plant defenses against *M. persicae* [10,11,12]. Glucosinolates are herbivores-deterrent secondary metabolites in *Brassicaceae* that increase upon aphid feeding and reduce *A. thaliana* susceptibility to aphids [13,14,15]. Finally, callose deposition, modification of starch content, and stimulation of senescence constitute additional modifications that may affect *M. persicae* [7].

*Arabidopsis thaliana* can, among other plant viruses, be infected by poleroviruses (*Polerovirus* genus, *Luteoviridae* family) that are transmitted by aphids in a circulative and non-propagative mode [16]. Polerovirus particles are acquired by aphids during phloem sap ingestion on infected plants. Virions migrate through the gut and internalize into intestinal cells after binding to specific virus receptors [17]. Virus particles are then transported through the intestinal cells and released in the hemolymph. Finally, polerovirus particles cross the salivary gland cells and are released together with saliva into a new plant host during a subsequent feeding event [18].

There is growing evidence that aphid-transmitted viruses can affect plant phenotypes and vector behaviors in ways that may ultimately facilitate virus acquisition and inoculation [19,20]. This concept of plant and aphid “manipulation” by the virus also applies to viruses in the *Luteoviridae* family. Polerovirus infection induces leaf yellowing, which may be visually attractive for aphids [21]. The major viral determinant governing both symptoms expressions, including yellowing, and aphid transmission, is attributed to the minor capsid protein of poleroviruses [22,23]. These viruses also increase volatiles emission from infected plants that attract non-viruliferous aphids [24,25]. In addition, polerovirus infection alters plant palatability and quality that affect aphid feeding behavior and performances [26,27]. Changes in aphid behavior following virus acquisition are shown for *Cucurbit aphid-borne virus* (CABYV), *Potato leafroll virus* (PLRV) (*Polerovirus* genus, *Luteoviridae* family), and *Barley yellow dwarf virus* (BYDV) (*Luteovirus* genus, *Luteoviridae* family) [26,28,29]. Most studies conducted so far on potential “manipulations” of plants and aphids by poleroviruses and luteoviruses are mainly descriptive, and the molecular mechanisms behind the observed effects of the viruses on plants or on aphids are still unknown.

Knowledge on the plant response to poleroviruses is lacking, but transcriptomic analyses on plants infected by other aphid-transmitted viruses identified several plant defense pathways induced by viral infection. In addition to the RNA silencing pathway, these include stress and defense responses characterized by the induction of heat shock proteins and the accumulation of pathogenesis-related proteins mediated by salicylic acid [30,31,32]. Genes involved in plant growth and development like those modulating accumulation of abscisic acid, auxin, cytokinin, gibberellin, and ethylene are also induced [30,31,32]. Since common pathways or reactions are turned on following aphid infestation or virus infection, like the jasmonic acid pathway, response to hydrogen peroxide, and callose deposition, it is conceivable that plant defensive signaling and metabolism serve viruses to spread between plants by influencing aphid performance and feeding behavior. Until now, only a few viral proteins are supposed to impact aphid behavior (for review [33,34]). Some of these viral factors interfere with the jasmonic acid or ethylene pathways, or with the production of reactive oxygen species [33,34,35]. For example, it was shown that *Cucumber mosaic virus*, a non-persistently transmitted virus, drives accumulation of reactive oxygen species through the action of its 2b silencing suppressor, which consequently shortens aphid probes and increases virus acquisition [35]. Another advanced molecular study on viral impacts on aphid performance addresses the role of the nuclear inclusion-a protease (NIa-Pro) from *Turnip mosaic virus,* which regulates ethylene production and ethylene-related defense mechanisms [36,37,38]. The effect of NIa-Pro on aphid performance correlates with its ability to relocalize to the vacuole after aphid puncture [39].

Further functional studies are needed to identify metabolic pathways that are under virus control, influence aphid performance and/or feeding behavior, and ultimately impact virus acquisition and transmission. Consequently, we selected some *A. thaliana* mutants known to be affected in carbohydrates and glucosinolates metabolisms, phloem functions, and defense signaling and investigated their potential incidence on aphid traits, on *Turnip yellows virus* (TuYV, *Polerovirus* genus, *Luteoviridae* family) accumulation as well as on growth and feeding behavior of *M. persicae*, an efficient vector of TuYV. Mutants that influenced sap ingestion by aphids in TuYV-infected plants were further tested for their effect on virus transmission.

## 2. Materials and Methods

### 2.1. Plant Growth and Aphid Rearing

*Arabidopsis thaliana* mutant seeds were obtained from stock centers for *pad4-1* (PHYTOALEXIN DEFICIENT 4; N3806), *xth33* (xyloglucan:xyloglucosyl transferase 33; N16546), *ss3-2* (starch synthase 3; N869727). *quad* (*cyp79B2cyp79B3myb28myb29*) and *myc234* (*myc2myc3myc4*) were kindly provided by Philippe Reymond (Lausanne University, Lausanne, Switzerland). *atr1D* was obtained from Judith Bender (Johns Hopkins University, Baltimore, MD, USA) and *nata1* (GK-256F07) from Georg Jander (Boyce Thompson Institute, Ithaca, NY, USA). For *nata-1*, *xth33-1*, and *ss3-2 A. thaliana* mutants, homozygosis and T-DNA insertion were analyzed by PCR using primers described in Table S1. For *pad4-1* mutant, the point mutation was confirmed by *Bsm*F1 digestion of a PCR product amplified using the primers described in Table S1. *ATR1D* point mutation was confirmed by sequencing the PCR product encompassing the mutation (see primers in Table S1).

*A. thaliana* plants were grown in a growth chamber under 20 ± 1 °C and 14 h photoperiod under fluorescent lamps. Plants were aphid-inoculated two weeks post-sowing and kept in the same conditions. The *Myzus persicae* (Sulzer) (*Hemiptera*: *Aphididae*) clone originates from the Netherlands. Aphids were reared on Chinese cabbage (*Brassica rapa*) in a growth chamber under 20 ± 1 °C, and 16 h photoperiod.

### 2.2. Plant Inoculation with Viruliferous Aphids

*Arabidopsis thaliana* mutants and the reference ecotype Col-0 were inoculated using viruliferous *M. persicae*. Non-viruliferous aphids were fed with an artificial medium [22] containing TuYV at a concentration of 100 ng/µL and purified as described in [40]. After a 24 h acquisition period, two aphids were transferred onto each Col-0 plant or *A. thaliana* mutant. After 72 h inoculation, aphids were manually eliminated, and the plants were grown for 3 weeks before being tested by double-antibody sandwich enzyme-linked immunosorbent assay (DAS-ELISA) for infection status.

### 2.3. Virus Detection by DAS-ELISA and Quantitative RT-PCR (qRT-PCR)

TuYV was detected in non-inoculated leaves of *A. thaliana* by double-antibody sandwich enzyme-linked immunosorbent assay (DAS-ELISA) with a TuYV-specific polyclonal antiserum (Loewe) 3 weeks post-inoculation [41]. For qRT-PCR, total RNA was extracted 3 weeks post-inoculation using NucleoSpin RNA Plant Kit (Macherey-Nagel, Hœrdt, France). After quantification (Nanodrop 2000, ThermoFisher Scientific, Illkirch, France), 200 ng of viral RNA were converted to cDNA using the reverse primer BPqtR1 (Table S1) and the M-MLV reverse transcriptase kit (Promega, Charbonnières-les-Bains, France). The forward primer BPqtF0 and the reverse primer BPqtR1 (Table S1) were used to amplify the cDNA corresponding to nts 3694-3830 on TuYV genomic sequence (accession number NC_003743) using a CFX cycler (Biorad, Schiltigheim, France) programmed as follows: 3 min 95 °C, 40 amplification cycles for 10 s at 95 °C, 30 s at 60 °C. Melt curve analysis was performed from 60 °C to 95 °C with 5 s of 0.5 °C increments. Dilution series of 10^8^ to 10^3^ viral cDNA copies obtained from RNA extracted from purified virions were used for calibration. The comparison between the Ct values of the calibration standard and the samples provided an absolute quantification of TuYV genomes.

### 2.4. Plant to Plant Virus Transmission by M. Persicae

Non-viruliferous aphids (L4 and adults) were deposited onto TuYV-infected plants for virus acquisition during 24 h. After this acquisition access period, two aphids were deposited per Col-0 test plant for a 3-day inoculation period. Aphids were then eliminated by an insecticide treatment (Pirimor^®^, Certis Europe France, Guyancourt, France) and the test plants were assayed by DAS-ELISA 3 weeks later. In each virus transmission experiment, one to three virus source plants were used per condition and 20 to 60 test plants per condition.

### 2.5. Myzus Persicae Feeding Behavior Analysis on A. Thaliana Plants

Pools of synchronized first-instar nymphs were obtained from parthenogenetic adult females placed on detached leaves of pepper plant (*Capsicum annuum*) and deposited on 1.5% agar in Petri dishes during 24 h. Synchronized young adults were obtained 9–10 days later in the same device. The electrical penetration graph DC system [42] was used as described in [27]. The following parameters were extracted from the electropenetrography (EPG) recordings based on their characteristic EPG waveforms described in [43]: (Pr) stylet activity within plant tissues; (C) stylet pathways in plant tissues except phloem and xylem; (E1) salivation in phloem elements; (E2) passive phloem sap ingestion; (t > 1E2) time to first phloem ingestion; (G) active xylem sap ingestion; (F) derailed stylet mechanics. The feeding behavior was recorded for 8 h on infected or non-infected plants used only once.

### 2.6. Myzus Persicae Performance on A. Thaliana Mutants

*Myzus persicae* performance was evaluated by measuring the weight of individual aphids. Synchronized *M. persicae* first instar (L1 stage) was obtained as described above and deposited on whole plants of *A. thaliana* mutant (50 aphids on three different plants). After 10 days, thirty aphids were randomly selected and weighted individually with an electronic precision scale (Sartorius, MSE3.6P-000-DM, maximum 3.1 Kg, low 1 μg, (dd) = 1 μg).

### 2.7. Statistical Analyses

Data on TuYV accumulation in mutants and Col-0 are not normally distributed, thus we carried out a generalized linear model (GLM using quasi-Poisson distribution (link:log) followed by pairwise comparisons using estimated marginal means (package R: ‘emmeans’).

We used a generalized linear model (GLM) with a likelihood ratio and Chi-squared test to assess whether there was an effect of TuYV infection, or an effect of the mutation introduced into *A. thaliana* genome on *M. persicae* performance and feeding behavior. Data on aphid weight were analyzed by a GLM using Gaussian distribution. When a significant effect of one of the main factors was detected or when an interaction between factors was significant, a pairwise comparison using least-squares means (package R: “lsmeans”) (*p*-value adjustment with Tukey’s method) at the 0.05 significance level was used to test for differences between treatments. Data on aphid feeding behavior (s-E2) are not normally distributed, accordingly we carried out a GLM using a gamma (link = ”inverse”) distribution. When a significant effect of one of the main factors was detected or when an interaction between factors was significant, a pairwise comparison using least-squares means (package R: “lsmeans”) (*p*-value adjustment with Tukey’s method) at the 0.05 significance level was used to test for differences between treatments. Data on aphid feeding behavior (t1 < E2) were modeled using the Cox proportional hazards (CPH) model and we treated cases where the given event did not occur as censored. The assumption of validity of proportional hazards was checked using the functions “coxph” and “cox.zph”, respectively (package R: “survival”). The fit of all generalized linear models was controlled by inspecting residuals and QQ plots.

Comparisons of transmission efficiencies were analyzed by Pearson’s Chi-squared tests and followed by pairwise comparisons using Pearson’s Chi-squared tests with Yates’ continuity correction (*p*-value adjustment method: fdr) (package R: “RVAideMemoire”).

All statistical analyses were performed using the R software version 3.3.2 (The R Foundation, https://www.r-project.org/).

## 3. Results

### 3.1. TuYV Infection Assay of the A. thaliana Mutant Lines

We selected mutant lines of *A. thaliana* affected in various metabolic pathways expected to influence aphid fitness and behavior. Some effects of the mutations on *M. persicae* were previously reported (Table 1). Two mutants were modified in the carbohydrate metabolism. *xth33* bears a mutation in a gene involved in cell wall remodeling and *ss3-2*, which overaccumulates starch, a defense mechanism against *M. persicae* [44,45]. The *nata1* mutant contains a mutation in a gene controlling the synthesis of a non-protein amino acid, N^δ^-acetylornithine, which negatively impacts *M. persicae* when aphids acquire it from artificial medium or from plant leaves transiently expressing NATA1 [46]. Glucosinolates are secondary metabolites that have a defensive function against herbivorous insects. In particular, indole glucosinolates are reported to have a detrimental effect on aphids [14]. We tested three mutant lines affected in this pathway, *myc234*, *quad,* and *atr1D*. *myc234* contains mutations in three transcription factors regulating jasmonic acid responses (MYC2, MYC3, and MYC4) [47] and the *quad* mutant contains mutations in two cytochrome P450s genes (CYP79B2 and CYP79B3) and two transcription factors, MYB28 and MYB29, resulting in very low glucosinolates containing plants [48,49]. Therefore, *myc234* and *quad* mutants accumulate low levels of glucosinolates. In contrast, the overexpression of the MYB transcription factor ATR1, in the *atr1D* mutant, results in increased accumulation of indole glucosinolates [50]. *PAD*4 gene is part of a defense regulatory pathway against various pests and pathogens including *M. persicae* and the mutation in the *pad4-1* mutant results in a phytoalexin-deficient mutant, which positively affects aphid performance and phloem activity [51,52]. None of the mutated plants exhibit abnormal growth and development when compared to Col-0.

**Figure 1:**
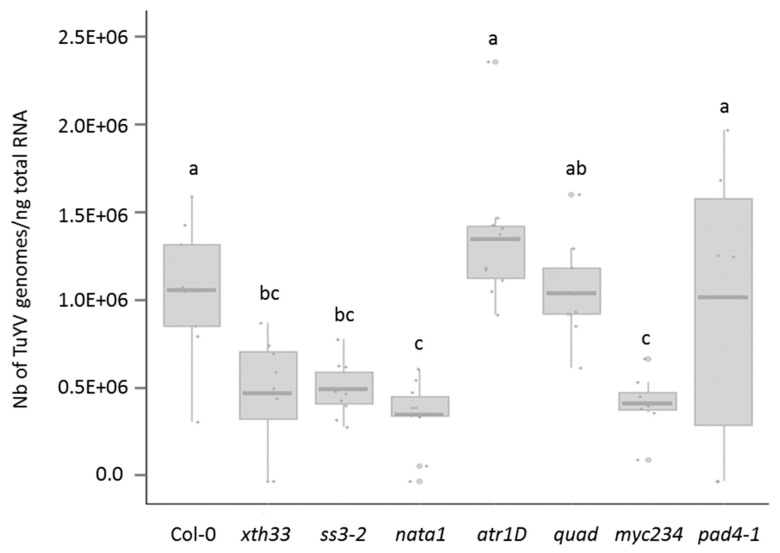
TuYV accumulation in *A. thaliana* mutant lines is assessed by qRT-PCR. The number of TuYV genome copies are normalized to ng of total plant RNA. This graph represents one experiment in which all plants are grown simultaneously and inoculated with viruliferous aphids from the same batch. Each dot corresponds to a plant sample. Box plots show median (line), 25%–75% percentiles (box) and 10%–90% percentiles (whiskers). TuYV accumulation varies with *A. thaliana* mutant lines (generalized linear model (GLM), df = 64, F = −10.38, *p* < 0.001). Letters show significant differences between plants as tested with a GLM followed by pairwise comparisons using estimated marginal means (Tukey’s method). Eight to ten plants per accession are tested in technical triplicates. Each point corresponds to the mean of the triplicates.

The capacity of TuYV to infect the different mutant lines was addressed by inoculating young plants with viruliferous aphids. Since TuYV-infected Col-0 do not develop symptoms [57], all aphid-inoculated plants were assayed by DAS-ELISA three weeks later. All mutants became infected with TuYV suggesting that none of the mutations had a strong detrimental effect on TuYV infection (Table S2). TuYV accumulation was then measured by qRT-PCR on DAS-ELISA positive plants. Compared to Col-0, virus titers were similar in *atr1D*, *quad*, and *pad4-1* and lower in *xth33-1*, *ss3-2*, *nata1*, and *myc234* infected plants (Figure 1).

### 3.2. TuYV Transmission Efficiency by M. persicae from Infected atr1D, quad, myc234, and pad4-1 Mutants

In order to address whether TuYV titers in infected plant influence virus acquisition and transmission, TuYV transmissibility by aphids was analyzed using several infected mutant plants as the virus source: atr1D, quad, and pad4-1 in which virus accumulation was similar to Col-0 and myc234 which display a lower virus titer compared to Col-0 (Figure 1). Four virus transmission experiments by aphids were performed. In three experiments, the virus acquisition period was 24 h (Table 2, Exp. 1, 2, and 3) which is a duration commonly used in TuYV transmission assays [22]. A significative reduction in TuYV transmission efficiency was only observed when atr1D was used as the virus source (Table 2, Exp. 1 and 2). The fourth transmission experiment was conducted using a shorter virus acquisition period of 8 h to evaluate if the impact of a mutation was more visible by reducing the acquisition time (Table 2, Exp. 4). This experiment confirms the lower TuYV transmission efficiency when infected atr1D plants are used as virus source but in addition, a decreased transmission rate is observed when aphids feed on infected pad4-1 plants for 8 h and not for 24 h. The virus transmission efficiency is therefore not correlated to the virus accumulation in the plants since both mutants (atr1D and pad4-1) sustained similar levels of TuYV when compared to Col-0 (Figure 1). TuYV transmission efficiencies from infected quad or myc234 mutants are not significantly different from those of infected Col-0 plants (Table 2, Exp. 1, 2, 3 and 4) despite a reduced virus accumulation in myc234 (Figure 1).

In all four independent experiments, virus titers in atr1D, quad, myc234, and pad4-1 plants were not correlated to the virus transmission efficiency suggesting that additional factors influence virus uptake from the infected mutated plants.

### 3.3. Effect of the Mutations and TuYV Infection of A. thaliana on Phloem Feeding Activity of M. persicae

Considering that the phloem sap ingestion phase is a crucial step for virus acquisition and therefore transmission of the phloem-limited TuYV, we measured, using electropenetrograpghy (EPG), the duration of sap ingestion (s_E2). Additionally, the time aphids took to perform the first phloem sap ingestion phase (t > 1E2), was analyzed as this parameter reflects plant palatability and might also affect TuYV acquisition.

On non-infected plants, total duration of phloem sap ingestion on ss3-2 and myc234 mutants is longer relative to nata1 and the time until the first phloem sap ingestion is longer on nata1 when compared to myc234 (Table 3). In TuYV-infected plants, the phloem sap ingestion is longer on xth33 and myc234 relative to atr1D and pad4-1 mutants (Table 3). In infected plants, the time to first phloem sap ingestion is higher in nata1 and atr1D relative to xth33, ss3-2, and myc234 (Table 3).

These results suggest that the reduced phloem sap ingestion phase on infected atr1D and pad4-1 during the 8 h of EPG recording could be responsible for the lower virus transmission efficiency observed when infected mutant plants are used as virus source (Table 2). Similarly, the longer sap ingestion phase on infected myc234 could compensate the lower virus titer in this mutant and explain why TuYV is transmitted as efficiently from this mutant than from infected Col-0 plants (Table 2).

No significant differences are observed in the total duration of the phloem sap ingestion between non-infected and infected plants (mutants and the reference Col-0) (Table 2). In contrast, the time to reach the phloem is reduced in infected xth33 plants when compared to non-infected plants and increased in infected atr1D and quad plants when compared to corresponding non-infected mutant plants (Table 2).

### 3.4. Biomass of Aphids Raised on Non-Infected and TuYV-Infected A. thaliana Mutant Lines

**Figure 2:**
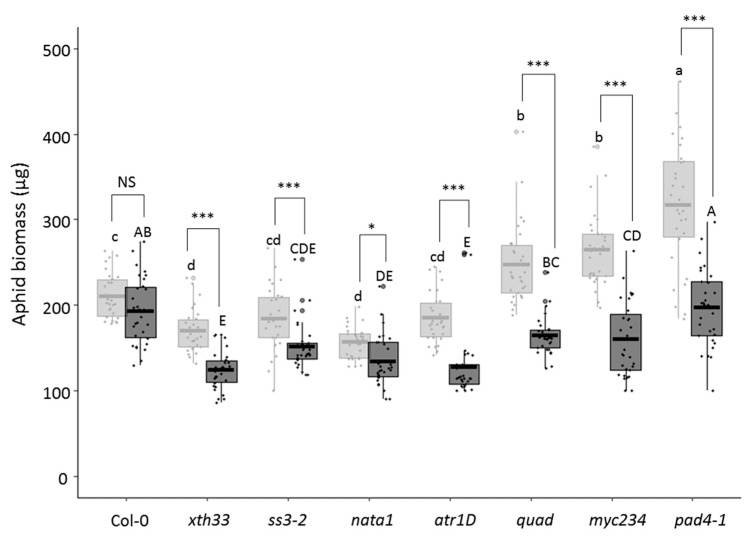
*Myzus persicae* biomass 10 days after feeding on non-infected (light grey) or TuYV-infected (dark grey) *A. thaliana* mutants, or on wild-type Col-0. Box plots show median (line), 25%–75% percentiles (box), and 10%–90% percentiles (whisker). Aphid biomass varied with the *A. thaliana* mutant lines and infection status (GLM, *p* < 0.001 for both factors). The asterisks indicate significant biomass differences of aphids raised on TuYV-infected and non-infected plants of the same mutant line (* *p* < 0.05, *** *p* < 0.001; NS, not significant). Letters indicate significant differences between plant genotypes obtained by GLM analysis followed by pairwise comparisons using least-squares means (lowercase letters for non-infected plants, capital letters for TuYV-infected plants).

Considering that the phloem sap ingestion phase is altered on several infected mutants, we analyzed whether these changes affect aphid biomass. We therefore analyzed the effect of *A. thaliana* mutations on aphid biomass, combined or not with TuYV infection. On non-infected plants and compared to Col-0, aphid biomass on *ss3-2* and *atr1D* is not significantly different while the aphid weight is lower on *xth33* and *nata1* and higher on *quad*, *myc234,* and *pad4-1* (Figure 2, light gray boxes). On TuYV-infected plants, the aphid biomass is not significantly different from the one on infected Col-0 for *quad* and *pad4-1* but it is lower on *xth33*, *ss3-2*, *nata1*, *atr1D,* and *myc234* (Figure 2, dark grey boxes). Except for the *atr1D* mutant for which a lower sap ingestion phase is observed together with a reduction in the aphid biomass when compared to Col-0, there is no correlation between the duration of the sap ingestion and the biomass for aphids fed on the other mutants.

Interestingly, when we compared the aphid weight between non-infected and TuYV-infected plants reared on the same *A. thaliana* line, a significant decrease is observed for all mutants while no difference is seen between infected and non-infected Col-0 plants (Figure 2, compare light and dark boxes).

## 4. Discussion

In this study, we addressed the effect of mutations in the *A. thaliana* genome on accumulation and aphid transmission of the phloem-limited TuYV, but also on aphid vector biomass and feeding behavior. The mutants are affected in different metabolic pathways and selected based on previous reports highlighting physiological and behavioral effects on aphids. This study is the first to address the combined effect of specific mutations in *A. thaliana* and TuYV infection on different aphid traits, biomass, and feeding activity. Two parameters related to phloem feeding phases were specifically selected, the total phloem sap ingestion duration and the time to the first sap ingestion phase, since both of them can potentially influence TuYV acquisition.

We observed that neither aphid performance nor feeding parameters are affected by TuYV infection in the wild-type context (Col-0). In contrast, in all mutants, the combined effect of the mutation and the infection effect aphid biomass, and in some of them, an additional effect on the feeding activity. This indicates that the virus infection interferes with several metabolic pathways that may impact aphid growth and feeding activity, as developed below. In the wild-type context (Col-0), the impact of the virus infection on several metabolic pathways can induce opposite effects on the aphids, which ultimately can result in a neutralization of these effects and no visible consequences on insect feeding or fitness parameters.

### 4.1. Effect of the Mutations and TuYV Infection on Aphid Feeding Activity

The *XTH33* gene encodes a xyloglucan:xyloglucosyl transferase involved in cell wall remodeling. In a previous report, *M. persicae* preferred settling on *A. thaliana xth33* mutant [44]. In our test conditions, aphids tended to feed more and reach the phloem faster on *xth33*, but only when the plants were infected. This suggests that in a non-mutated context (Col-0), and upon TuYV infection, this metabolic pathway could hinder aphid access to the phloem and reduce virus acquisition. We postulate that knocking out this pathway alleviates its negative effect on aphid feeding in infected plants. The second mutant in the carbohydrate metabolism is *ss3-2*, which overaccumulates starch and reduces *M. persicae* populations [53]. Reared on this mutant, aphids are faster in reaching the phloem vessels but only in infected plants. This suggests that overaccumulation of starch is not a barrier to stylet progression to the phloem.

In *A. thaliana nata1* mutant, which is unable to synthesize the non-protein amino acid N^δ^-acetylornithine [46], the phloem sap composition is altered. This non-proteinaceous amino acid is likely not metabolized by aphids. This compound is synthesized upon *M. persicae* infestation and has an in vitro toxic effect on sap-feeding insects including *M. persicae* [46]. We expected a beneficial effect (i.e., increased sap ingestion) on aphids feeding on *nata1* because of the absence of this aphid defensive compound, but no such effect was observed. Surprisingly, in the infected *nata1*, the time to perform the first phloem phase increased, revealing a beneficial effect of this component on aphids in Col-0.

The effect of glucosinolates is addressed in this study using three *A. thaliana* mutants, which accumulate different levels of these compounds. Glucosinolates are secondary metabolites that are hydrolyzed into toxic compounds after tissue damage of generalist herbivores. They affect not only herbivores by also their natural enemies [58,59]. The over-expresser mutant *atr1D* accumulates higher amounts of indole glucosinolates [50] while *myc234* and *quad* mutants sustain very low levels of glucosinolates [48,49]. The *myc234* mutant is practically devoid of glucosinolates and only traces of two indole glucosinolates remain [48]. It was previously reported that the genetic suppression of MYC function in the *myc234* mutant increases aphid attraction by releasing different volatiles and potentially also by modifying composition of plant metabolites [54]. In the *quad* mutant, the synthesis of both indole and aliphatic glucosinolates is impaired, but no impact on *M. persicae* population growth was reported [49,55]. Although *M. persicae* should not be in contact with vacuolar glucosinolates-hydrolyzing myrosinases when ingesting sap, indole glucosinolates are nonetheless degraded in the aphids and this degradation may be responsible for the deterrent effect of these compounds on aphids [14]. Supporting the role of indole glucosinolates in crucifer defense against aphids is their over-accumulation upon aphid infestation [13,15]. Our data showed a beneficial effect of low glucosinolates content only in *myc234* plants in which aphids reach the phloem faster and feed longer both in non-infected and infected conditions. In accordance with the overaccumulation of indole glucosinolates in *atr1D*, access to the phloem and sap ingestion are both negatively impacted but only in infected plants.

*PAD4* is a central player in the signaling defense pathway against pathogens including aphids, and *PAD4* expression in *A. thaliana* is stimulated by *M. persicae* infestation [51]. In the phytoalexin-deficient mutant *pad4-1* background, the aphid-induced senescence is inhibited resulting in a better aphid settling, feeding, and fecundity [12,52]. In contrast to these reports, we observed a reduced sap ingestion phase on *pad4-1*. The discrepancy in the results may be due to intrinsic differences in the aphid clones, but it is also conceivable that the increased sap ingestion observed on *pad4-1* by Pegadaraju et al. [12] is due to an abnormal low phloem feeding activity of *M. persicae* on the reference Col-0 in this study. In our study, we observed an opposite effect of the mutation (reduced sap ingestion phase) but only upon virus infection.

### 4.2. Effect of the Mutations and TuYV Infection on Aphid Biomass

Aphid biomass on the *A. thaliana* mutants was not addressed before. As expected, *M. persicae*’s growth increases on non-infected *pad4-1*, which accumulates a low content of phytoalexins, and on *quad* and *myc234*, which are substantially deprived of glucosinolates. The aphid biomass on the other mutants is not related to the biological function of the mutated genes. However, regardless of the mutation in the *A. thaliana* genome, we always observe a reduction of the aphid biomass on infected mutant plants when compared to the non-infected mutants. This effect on aphid biomass was not clearly observed on Col-0 despite a slight, but not significant, reduction in aphid size. One hypothesis to explain this result is an additive effect of the virus infection and the mutations on aphid development. TuYV infection, together with the mutations in the different metabolic pathways, likely affect sap nutrient value and therefore aphid biomass. In this regard, the constitutive expression of the P4 protein from *Potato leafroll virus* (PLRV, *Polerovirus*, *Luteoviridae*) is reported to induce changes in carbohydrate status which could have consequences on the aphid biomass [60].

It is interesting to point out that the aphid weight measured on non-infected or infected mutants is not correlated to the duration of the sap ingestion. Indeed, no statistical differences in the sap ingestion phase were observed between non-infected and infected mutants whereas a significant reduction in the aphid biomass was visible with all infected mutated plants. This strongly suggests that nutritional quality of phloem sap contributes more to aphid biomass than the volume of sap ingested by aphids.

### 4.3. Impact of the Mutations on TuYV Transmission by Aphids

The impact of the mutations in *atr1D*, *quad*, *myc234*, and *pad4-1* on TuYV transmission efficiency is addressed. Only the *atr1D* mutation has a reproducible negative effect on TuYV transmission by aphids regardless of the time of virus acquisition (8 or 24 h), while it has no effect on virus accumulation. A slight decrease in the virus transmission rate is also observed with *pad4-1* but only when the acquisition time is 8 h. The reduced sap ingestion phase observed during the 8 h of recording on infected *atr1D* and *pad4-1* could be responsible for a lower virus uptake, and subsequently, a reduced TuYV transmission. It is conceivable that the decreased sap ingestion observed on *pad4-1* during the first eight hours of feeding is transient and that the expected beneficial effect of the absence of phytoalexins in this mutant sets in only later. This delay would explain why an effect on virus transmission was observed after a virus acquisition period of 8 h but not 24 h. In the case of *atr1D,* it is also possible that the higher content of indole glucosinolates influences virus uptake, internalization into intestinal cells or even virus inoculation into test plants. Detection of plant glucosinolates in aphids [61] suggests that they could exert their negative effect also in the aphid’s body by altering virus transport from the digestive tube to the accessory salivary glands.

Lastly, our study also suggests that the time required to perform the first phloem sap ingestion phase, which is extended in *atr1D,* could be a more important factor for virus uptake than expected. Hindrance during phloem access could discourage aphids to ingest sap as they normally do, and this could limit virus acquisition.

## 5. Conclusions

**Figure 3:**
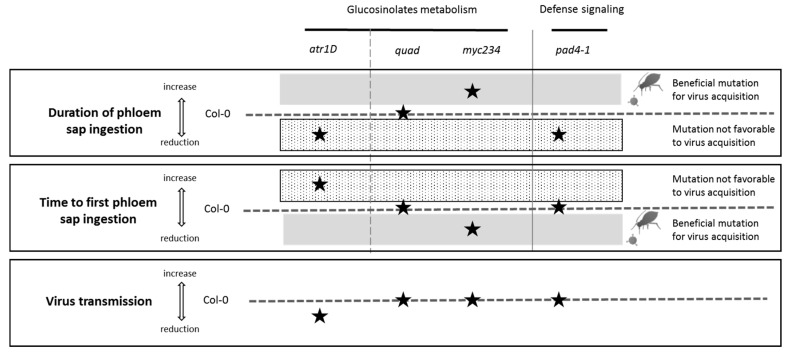
Schematic representation of the combined effect of *A. thaliana* mutations and TuYV infection on phloem sap ingestion, time until the first sap ingestion phase, and virus transmission. Black stars stand for the effect of the mutation in TuYV-infected *A. thaliana* when compared to the reference Col-0 represented by a dashed line. When the stars are above the line, the parameter values observed for the mutants are higher than those for Col-0 and when the stars are below the line, the values are lower than those for Col-0. In grey is a zone that potentially represents beneficial conditions for virus acquisition (long sap ingestion and short delay to perform the first sap ingestion phase). The dotted line area symbolizes a zone not favorable for virus acquisition with reduced sap ingestion and increased time until the first sap ingestion phase.

In this work, we addressed the effect of different mutations affecting several plant biological pathways known to influence aphid performance and behavior. We observed that virus infection and most mutations have cumulative effects and induce a biomass reduction of aphids suggesting that the deregulations triggered by TuYV infection also impact the biological pathways targeted by the mutations. It is however conceivable that the infection activates plant defense responses which, in combination with the mutations, affects aphid traits. The mutations in *myc234* result in an increased phloem sap ingestion and a faster phloem access, but no beneficial effect on virus transmission was observed in our experimental conditions, maybe because of the low virus accumulation in this mutant. A completely adverse situation was observed in *atr1D* mutant with reduced sap ingestion and a delayed phloem access leading to a decreased virus transmission (Figure 3). This result highlights a negative effect of the overexpression of *ATR1D* on virus transmission. It is interesting to point out that expression of this gene is reduced in TuYV-infected Col-0 that are infested for 6 h with non-viruliferous aphids while no such deregulation was observed in plants solely infected with the virus or solely infested with aphids (Krieger and Ziegler-Graff, unpublished). Such gene downregulation in the condition of virus infection and aphid infestation could indicate a plant manipulation by the virus upon aphid infestation. However, this assumption requires extensive studies to be confirmed. This work points the way towards the identification of metabolic pathways which could be potential targets to reduce virus acquisition by aphids, and as a consequence, virus propagation among plants.

## Figures and Tables

**Table 1:** *Myzus persicae* fitness and behavior on the *A. thaliana* mutants used in this study.

Gene Number	Mutant	Gene Name	Effects on *M. persicae* Observed on the Corresponding Mutant	References
At1g10550	*xth33*	xyloglucan:xyloglucosyl transferase33	Settling preference.	[44]
At1g11720	*ss3-2*	starch synthase III	Reduced population.	[45,53]
At2g39030	*nata1*	Gcn5-related N-acetyltransferase	Reduced aphid progeny when N^δ^-acetylornithine was added to an artificial medium or when transiently produced in leaves. No effect on aphids when *NATA1* is expressed in transgenic plants.	[46]
At1g32640 At5g46760 At4g17880	*myc234*	MYC2, MYC3, MYC4	Higher aphid attractiveness.	[47,54]
At2g22330 At4g39950 At5g61420 At5g07690	*quad*	glucosinolate quadruple mutant	No increase of aphid population.	[49,55]
At5g60890	*atr1D*	altered tryptophan regulation 1	Decreased aphid reproduction at the rosette stage. No increase of aphid population.	[50,55,56]
At3g52430	*pad4-1*	phytoalexin-deficient 4	Better feeding activity and enhanced susceptibility to aphids.	[12,52]

**Table 2:** TuYV transmission efficiency from infected *A. thaliana* mutants atr1D, quad, myc234, and pad4-1.

Virus Source Plant	Exp. 1 ^1^	Exp. 2 ^2^	Exp. 3 ^2^	Exp. 4 ^3^
***atr1D***	23/60 ^4^ **(38.33%)** a ^5^	26/45 (57.70%) a	/	41/60 **(68.33%)** a
***Quad***	43/60 (71.67%) b	41/45 (91.11%) b	/	48/60 (80.00%) b
***myc234***	/	/	16/20 (80.00%)	
***pad4-1***	43/60 (71.67%) b	/	/	42/60 **(70.00%)** a
***Col-0***	49/60 (81.67%) b	42/43 (93.33%) b	18/20 (90.00%)	53/60 (88.33%) b

^1^ The virus titer of the source plants is presented in Table S3. ^2^ The virus titer of the source plants is presented in Table S4. ^3^ The virus titer of the source plants is presented in Table S5. ^4^ Number of infected test plants (Col-0) assayed by DAS-ELISA/total number of plants inoculated with viruliferous aphids. A plant is considered infected when the OD (optical density) value of the leaf extract is above twice the mean OD values of three non-infected plants plus three times the standard deviation of these values. ^5^ Letters indicate significant differences between plant genotypes obtained by Pearson’s Chi-squared tests (χ^2^ = 28.67, df = 3, *p* < 0.001; χ^2^ = 27.52, df = 2, *p* < 0.001; χ^2^ = 0.20, df = 1, *p* = 0.658; and χ^2^ = 0.76, df = 3, *p* = 0.033 for experiments 1, 2, 3, and 4, respectively) followed by pairwise comparisons.

**Table 3:** Feeding behavior of *M. persicae* on non-infected and TuYV-infected *A. thaliana* mutants.

		Col-0	*xth33*	*ss3-2*	*nata1*	*atr1D*	*quad*	*myc234*	*pad4-1*
**s_E2 (min) ^2^**	*n* ^1^	19	18	20	18	21	20	22	20
	**non-inf.**	199.54 ± 30.02 ab	254.72 ± 33.76 ab	**274.05 ± 30.95 a**	**129.09 ± 23.14 b**	183.04 ± 27.83 ab	292.55 ± 26.18 ab	**277.90 ± 20.16 a**	165.82 ± 24.88 ab
	**TuYV-infected**	229.09 ± 30.74 AB	**268.44 ± 27.88 A**	227.06 ± 23.97AB	182.61 ± 36.73 AB	**139.60 ± 33.79 B**	154.84 ± 28.87 AB	**265.82 ± 26.07 A**	**132.28 ± 24.63 B**
	**non-inf. versus inf.**	ns	ns	ns	ns	ns	ns	ns	ns
**t > 1E2 (min) ^3^**	*n*	18	22	20	17	18	20	20	20
	**non-inf.**	167.14 ± 23.47 ab	134.49 ± 35.50 ab	104.73 ± 16.21 ab	**181.67 ± 31.40 a**	179.15 ± 22.98 ab	108.19 ± 21.26 ab	**130.94 ± 17.95 b**	167.43 ± 28.20 ab
	**TuYV-infected**	162.55 ± 25.99 AB	**105.71 ± 14.00 B**	**131.53 ± 25.95 B**	**287.20 ± 31.67 A**	**218.47 ± 36.48 A**	218.12 ± 39.56 AB	**108.96 ± 17.86 B**	108.34 ± 25.38 AB
	**non-inf. versus inf.**	ns	*	ns	ns	*	**	ns	ns

^1^ n: number of individuals for which the parameter was recorded. ^2^ s_E2: total duration of phloem sap ingestion. Phloem sap ingestion varies according to the A. thaliana mutant lines (GLM, *p* < 0.001) but not the infection status (*p* > 0.05). Letters indicate significant differences between plant genotypes obtained by GLM analysis followed by pairwise comparisons using least-squares means (lowercase letters for non-infected plants, capital letters for TuYV-infected plants). ^3^ t > 1E2: time to first phloem sap ingestion. Time to the first phloem sap ingestion varies according to the A. thaliana mutant lines (Cox model, *p* < 0.001) but not the infection status (*p* = 0.33). Letters indicate significant differences between plant genotypes obtained by GLM analysis followed by pairwise comparisons using least-squares means (lowercase letters for non-infected plants, capital letters for TuYV-infected plants). Dark grey boxes highlight significant different values of parameters with a potential beneficial effect on virus acquisition and light grey boxes highlight significant different values of parameters with a potential negative effect on virus acquisition. The asterisks indicate a significant difference between TuYV-infected and non-infected plants (* *p* < 0.05, ** **p** < 0.01; ns, not significant).

## Supplementary Materials

The following are available online at https://www.mdpi.com/1999-4915/12/2/146/s1, Table S1: Primer sequences; Table S2: TuYV infection of *A. thaliana* mutant lines; Table S3: TuYV titer measured by qRT-PCR in infected *A. thaliana* mutants *quad*, *pad4-1*, and *atr1D* plants used as virus source in a virus transmission experiment (Exp. 1, Table 3); Table S4: TuYV accumulation measured by ELISA in infected *A. thaliana* mutants *quad*, *atr1D,* and *myc234* plants used as virus source in virus transmission experiments (Exp. 2 and 3, Table 3); Table S5: TuYV titer measured by qRT-PCR in infected *A. thaliana* mutants *quad*, *pad4-1*, and *atr1D* plants used as virus source in a virus transmission experiment (Exp. 4, Table 3).
