# Supplementary material for: Impact of Mutations in Arabidopsis thaliana Metabolic Pathways on Polerovirus Accumulation, Aphid Performance, and Feeding Behavior
Source: Viruses. 2020 Jan 27;12(2):146. doi: 10.3390/v12020146 (PMC7077285; doi:10.3390/v12020146)
Supplement: Supplementary file 1 [file viruses-12-00146-s001.zip › Table S1 Bogaert et al Viruses.pdf]

Table S1 : Primer sequences

A. thaliana mutant	Germplasm name	Mutated gene	Primer sequences used to detect mutation		Primer sequences used to detect wild type allele	
xth33	CS16546	AT1G10550	Sense	GAACAGCCACTTAACTCCACG	Sense	GAACAGCCACTTAACTCCACG
			Antisense	TGGTTCACGTAGTGGGCCATCG	Antisense	CAGTGCAAGACTCAAGCTTCC
nata1	GK256F07	AT2G39030	Sense	GGGCTACACTGAATTGGTAGCTC	Sense	ATTTTCCAAGAAGAGGGATCG
			Antisense	ACTTTAAAGGACCGATCGCTC	Antisense	ACTTTAAAGGACCGATCGCTC
pad4-1	N3806	AT3G52430	Sense	TAGCTACCAAGCTGGTGTTGCGTTAG	Sense	TAGCTACCAAGCTGGTGTTGCGTTAG
			Antisense	CATTTTGCACTTGAACTCTTTCAGATTC	Antisense	CATTTTGCACTTGAACTCTTTCAGATTC
atr1D	1005959182	AT4G24520	Sense	CGGACAAGAGCATCAGAAC		
			Antisense	GAGAATTGAATAGAGCGTCTG		
