# Supplementary material for: Impact of Mutations in Arabidopsis thaliana Metabolic Pathways on Polerovirus Accumulation, Aphid Performance, and Feeding Behavior
Source: Viruses. 2020 Jan 27;12(2):146. doi: 10.3390/v12020146 (PMC7077285; doi:10.3390/v12020146)
Supplement: Supplementary file 1 [file viruses-12-00146-s001.zip › Table S2 Bogaert et al Viruses.pdf]

Table S2. TuYV infection of *A. thaliana* mutants lines.

***A. thaliana* line**	nb inf. plants/total inoc. **(% inf. plants)**[1]
**Col-0**	132/167 **(79%)**
xth33-1	45/60 **(75%)**
ss3-2	48/75 **(64%)**
nata1	24/46 **(52%)**
atr1D	19/25 **(76%)**
quad	125/146 **(86%)**
myc234	71/97 **(73%)**
pad4-1	48/66 **(73%)**

[1]Thirteen independent experiments have been performed. The ratios represent the sum of the number of infected plants/total plants inoculated with viruliferous aphids. In brackets, the percentage of infected plants is indicated. A plant is considered infected when the OD value of the DAS-ELISA test is above twice the mean OD values of three non-infected plants and three times the standard deviation of these values.
