# Supplementary material for: Impact of Mutations in Arabidopsis thaliana Metabolic Pathways on Polerovirus Accumulation, Aphid Performance, and Feeding Behavior
Source: Viruses. 2020 Jan 27;12(2):146. doi: 10.3390/v12020146 (PMC7077285; doi:10.3390/v12020146)
Supplement: Supplementary file 1 [file viruses-12-00146-s001.zip › Table S3 Bogaert et al Viruses.pdf]

**Table S3.** TuYV titer measured by qRT-PCR in infected *A. thaliana* mutants *quad*, *pad4-1* and *atr1D* plants used as virus source in a virus transmission experiment (Exp. 1 Table 3).

| ***A. thaliana* mutant plants** | **TuYV genome copies/ng total RNA ± SE[1]** | **Mean TuYV genome copies/ng total RNA ± SE** |
|---|---|---|
| ***quad* # 1** | 4.74E+05 ± 2.88E+04 | 4.43E+05 ± 1.58E+04 |
| ***quad* # 2** | 4.22E+05 ± 2.52E+04 | |
| ***quad* # 3** | 4.33E+05 ± 4.38E+04 | |
| ***pad4-1* # 1** | 4.35E+05 ± 3.84E+04 | 4.23E+05 ± 2.19E+04 |
| ***pad4-1* # 2** | 3.81E+05 ± 1.54E+04 | |
| ***pad4-1* # 3** | 4.54E+05 ± 1.42E+04 | |
| ***atr1D* # 1** | 7.37E+05 ± 2.70E+04 | 4.78E+05 ± 1.29E+05 |
| ***atr1D* # 2** | 3.43E+05 ± 9.45E+03 | |
| ***atr1D* # 3** | 3.54E+05 ± 2.92E+04 | |
| **Col-0 # 1** | 6.21E+05 ± 2.75E+04 | 6.15E+05± 2.35E+04 |
| **Col-0 # 2** | 6.53E+05 ± 1.49E+04 | |
| **Col-0 # 3** | 5.72E+05 ± 2.82E+04 | |

[1]TuYV genome copies in each plant sample analyzed by qRT-PCR ± Standard Error of technical triplicates.
