# Supplementary material for: Impact of Mutations in Arabidopsis thaliana Metabolic Pathways on Polerovirus Accumulation, Aphid Performance, and Feeding Behavior
Source: Viruses. 2020 Jan 27;12(2):146. doi: 10.3390/v12020146 (PMC7077285; doi:10.3390/v12020146)
Supplement: Supplementary file 1 [file viruses-12-00146-s001.zip › Table S4 Bogaert et al Viruses.pdf]

Table S4. TuYV accumulation measured by ELISA in infected *A. thaliana* mutants *quad, atr1D* and *myc234* plants used as virus source in virus transmission experiments (Exp. 2 & 3 Table 3).

**Experiment**[1]	**Virus source plant**	**DAS-ELISA OD of infected plants**[2]	**Mean OD +/- SE**[3]
**Exp. 2**	***quad* # 1**<br>***quad* # 2**<br>***quad* # 3**	0.57<br>0.62<br>0.59	0.59 ± 0.01
	atr1D* # 1**<br>atr1D* # 2**<br>***atr1D* # 3**	0.41<br>0.31<br>0.41	0.38 ± 0.03
	**Col-0 # 1**<br>**Col-0 # 2**<br>**Col-0 # 3**	0.44<br>0.45<br>0.45	0.45 ± 0.00
**Exp. 3**	***myc234* # 1**	1.24	/
	**Col-0 # 1**	1.82	/

[1]The experiments are those mentioned in Table 3.

[2]DAS-ELISA OD value at 405 nm 1 h after substrate incubation. The mean OD value of three non-infected Col-0 is 0.09 ± 0.005 for exp. 2 and 0.153 ± 0.004 for exp. 3.

[3]Mean DAS-ELISA OD value +/- Standard Error.
