# Supplementary material for: Impact of Mutations in Arabidopsis thaliana Metabolic Pathways on Polerovirus Accumulation, Aphid Performance, and Feeding Behavior
Source: Viruses. 2020 Jan 27;12(2):146. doi: 10.3390/v12020146 (PMC7077285; doi:10.3390/v12020146)
Supplement: Supplementary file 1 [file viruses-12-00146-s001.zip › Table S5 Bogaert et al Viruses.pdf]

Table S5. TuYV titer measured by qRT-PCR in infected *A. thaliana* mutants *quad*, *pad4-1* and *atr1D* plants used as virus source in a virus transmission experiment (Exp. 4 Table 3).

***A. thaliana* mutant plants**	**TuYV genome copies/ng total RNA ± SE[1]**	**Mean TuYV genome copies/ng total RNA ± SE**
quad **# 1**	3.23E+05 ± 2.01E+04	3.80E+05 ± 5.24E+04
quad **# 2**	4.85E+05 ± 6.11E+04	
quad **# 3**	3.33E+05 ± 1.56E+04	
pad4-1 **# 1**	3.82E+05 ± 1.79E+04	3.74E+05 ± 0.82E+04
pad4-1 **# 2**	3.58E+05 ± 1.82E+04	
pad4-1 **# 3**	3.83E+05 ± 2.98E+04	
atr1D **# 1**	4.39E+05 ± 2.62E+04	4.32E+05 ± 0.44E+04
atr1D **# 2**	4.24E+05 ± 2.10E+04	
atr1D **# 3**	4.34E+05 ± 1.36E+04	
**Col-0 # 1**	5.06E+05 ± 4.35E+04	3.70E+05± 6.82E+04
**Col-0 # 2**	2.92E+05 ± 2.02E+04	
**Col-0 # 3**	3.12E+05 ± 5.49E+03	

[1]TuYV genome copies in each plant sample analyzed by qRT-PCR ± Standard Error of technical triplicates.
